# Should there be a paradigm shift for the evaluation of isthmic thyroid nodules?

**DOI:** 10.1007/s40618-024-02313-6

**Published:** 2024-02-16

**Authors:** Yağmur Babayid, Asena Gökçay Canpolat, Atilla Halil Elhan, Koray Ceyhan, Demet Çorapçıoğlu, Mustafa Şahin

**Affiliations:** 1 Department of Internal Medicine, Ankara University School of Medicine, Ankara, Turkey; 2 Department of Endocrinology and Metabolism, Ankara University School of Medicine, Ankara, Turkey; 3 Department of Biostatistics, Ankara University School of Medicine, Ankara, Turkey; 4 Department of Pathology, Ankara University School of Medicine, Ankara, Turkey

**Keywords:** Isthmic nodules, Lobar nodules, Malignancy rate, Lymphovascular invasion

## Abstract

**Purpose:**

Although the thyroid isthmus seems like a rudimentary structure that connects bilateral lobes, it is an undiscovered area that needs to be explored. Currently, the data is evolving that the increase in the risk of malignancy is higher in the isthmic nodules, and extrathyroidal extensions and lymph node metastases are more common in isthmic-derived malignant thyroid nodules. Therefore, we aimed to compare the malignancy rate of isthmic and lobar nodules, the ultrasonographic features of isthmic and lobar nodules, and presence of lymph node metastases, distant metastases, and extrathyroidal invasions in malignant isthmic nodules.

**Methods:**

In this retrospective study, we enrolled patients between the ages of 18–80 years, who had thyroid nodule/nodules cytology and/or pathology results from January 2009 to November 2022. 9504 nodules were selected for the analysis of US findings, cytopathology results, and malignancy rates.

**Results:**

A mean ± SD age of 55.3 ± 13.0 years with a female to male ratio of [7618 (80.2%)/1886(19.8%)] were included in the study. 962 of the nodules were at isthmic localization; whereas 8542 nodules were at lobar localization. 1188 nodules were resulted as malignant from histopathological evaluation. Of the 1188 malignant nodules, 986 nodules were (83.0%) PTC, 114 nodules (9.6%) were FTC, 55 nodules were (4.6%) MTC, 16 nodules 1.3% were Hurtle cell carcinoma, 8 nodules (0.7%) were anaplastic thyroid carcinoma, and 9 nodules (0.8%) were thyroid tumors of uncertain malignant potential (TT-UMP). 156 of the malignant nodules (13.1%) were located in the isthmus, whereas the majority of the malignant nodules (n = 1032, 86.9%) were located at the lobar parts (right or left) of the thyroid. When the metastasis patterns of isthmic and lobar thyroid cancers were examined, no significant relationship was found between isthmic and lobar cancers in terms of capsule invasion (p = 0.435), muscle invasion (p = 0.294), and lymph node metastasis (p = 0.633). A significant relation was found between nodule localization (isthmus-upper-middle and lower lobes) and malignancy (p < 0.001). In our logistic regression analysis, isthmic and upper pole nodule localizations, age and TI-RADS were evaluated as independent risk factors for malignancy (p < 0.001 for all factors).

**Conclusion:**

We recommend nodule localization has to be considered an additional risk factor when performing a Fine Needle Aspiration Biopsy for the increased malignancy risk in this localization.

## Introduction

Although the thyroid isthmus resembles a rudimentary structure that connects the lower third of the right and left thyroid lobes, it remains an undiscovered area that requires exploration.

Among randomly selected individuals, the prevalence of thyroid nodules detected by high-resolution ultrasonography (US) is approximately 19–67%, and 5–15% of these are malignant [1, 2]. The rate of thyroid cancer diagnosis has been increasing with advanced US techniques and molecular testing; additionally, numerous guidelines have been developed to establish accurate diagnosis through fine-needle aspiration biopsy (FNAB) or surgical interventions. Based on data from current guidelines, the estimated risk for malignancy for nodules is between 7 and 15% [3]. Until now, no guideline has determined nodule localization as an indication for biopsy.

The prevalence of thyroid nodules located in the isthmus was found to be 4.2–6.4% [4]. Although previous studies have reported a lower frequency of cancer localised in the isthmus [1, 5], Papi et al. reported a frequency of 9% for malignant neoplasm of the solitary isthmic nodules. Moreover, they proposed that isthmic nodules behave similarly as their lateral thyroid lobe counterparts [6]. Recently, the data is evolving about the increase in the risk of malignancy is higher in the isthmic nodules, and extrathyroidal extensions and lymph node (LN) metastases are more common in isthmic-derived malignant thyroid nodules [7–9]. Therefore, more studies are required to determine the management of isthmic thyroid nodules and establish the optimal approach for thyroidectomy with/without prophylactic LN dissection. In addition, a different nodule approach may be required in newly developed nodule guidelines for isthmic thyroid nodules.

This study aimed to (i) compare the US features of isthmic and lobar nodules, (ii) compare the clinical and histopathological features and malignancy rates of isthmic and lobar nodules, and (iii) evaluate if any difference exists in LN metastases, distant metastases and extrathyroidal invasions in malignant isthmic nodules.

## Methods

### Patient selection

**Fig. 1:**
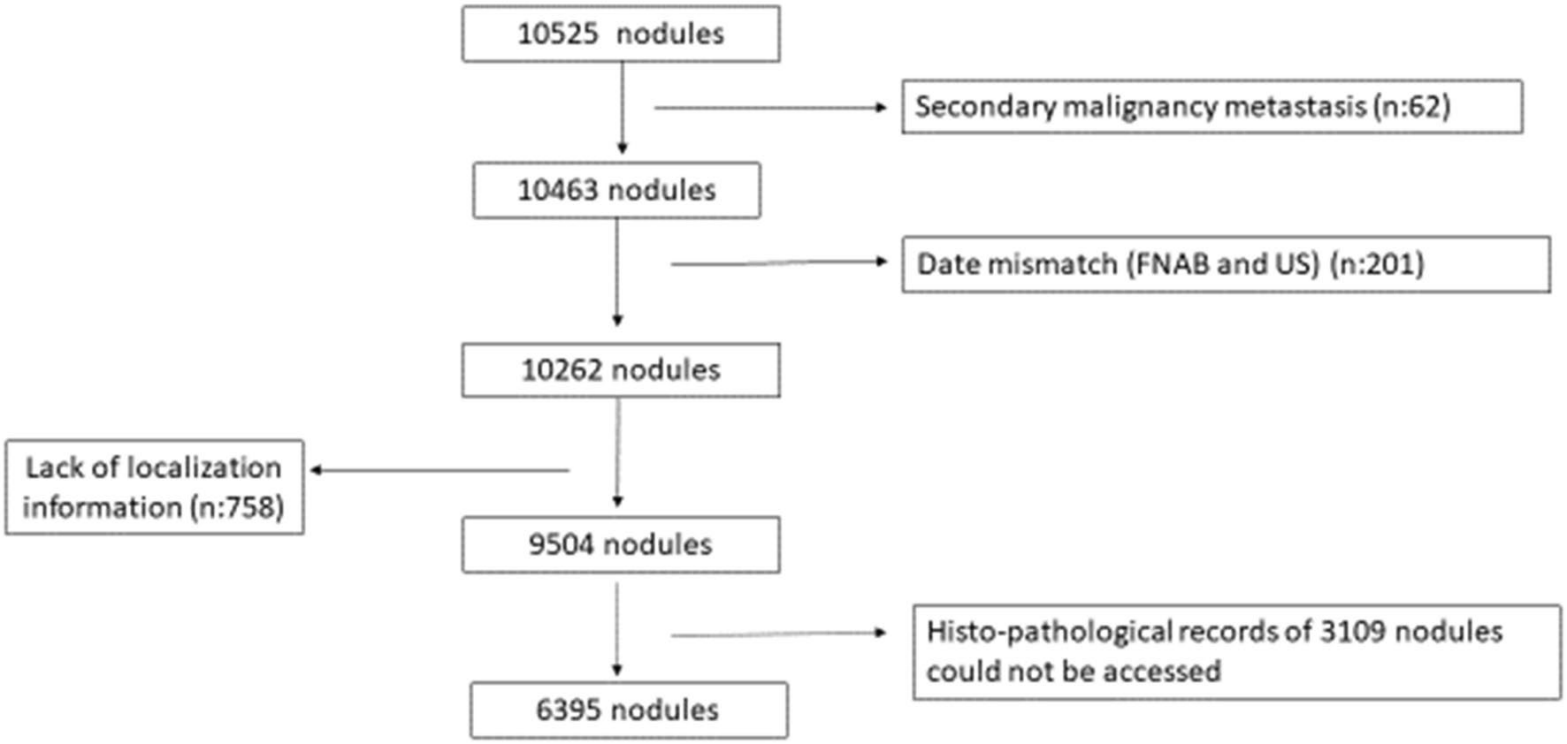
Inclusion diagram of the nodules with detailed ultrasound data and cytopathological and histopathological results

In this retrospective study, patients between the ages of 18 and 80 years, who had thyroid nodule cytology and/or pathology results in our tertiary Ankara İbn-i Sina Hospital from January 2009 to November 2022 were enrolled. The characteristics, location, and cytopathology results of 10525 nodules that were detected by thyroid US and evaluated via FNAB between 2009 and 2022 were examined, and the relationship between the characteristics, nodule location and malignancy rate were evaluated. Furthermore, surgically removed malignant nodules were investigated in terms of their metastasis sites (capsule, muscle, or LN) and micro-macrometastasis patterns. We also retrospectively compared patient characteristics, tumour size, extrathyroidal extension, lymphovascular and perineural invasion and patterns of LN metastasis in the isthmic and non-isthmic groups. During examination, the cytology report of 62 nodules were revealed as secondary cancer that had metastasised to the thyroid gland and was excluded from the study. A total of 201 nodules were excluded because of the mismatch between the date of FNAB and US. Also, 758 nodules were excluded because the age did not satisfy the inclusion criteria and due to lack of localization information either in the cytology or the US reports. As a result, from the US findings, cytopathology results, and malignancy rates, 9504 nodules were selected. After excluding 3109 nodules in which histopathological records could not be accessed. Of the total 6395 nodules, 1188 were determined as malignant.The nodules were included according to the nodule selection diagram (Fig. 1).

Age, sex, nodule localization (isthmus, right lobe and left lobe; upper portion, middle portion, and lower portion), nodule dimensions, nodule echogenicity (hypoechoic, isoechoic, and hyperechoic), nodule composition (solid, cystic, and mixed), margin irregularity, presence of microcalcification/macrocalcification, solitary/multinodular, nodule shape, maximum diameter, TIRADS-5, cytopathological results and histopathological results were examined for each patient. The paucity of the cases were multicentric and were located at the same lobes. We included the dominant tumor for the analyses of this study.

Thyroid US was performed by the same six experienced endocrinologists of the department who have the same technical education and uniformity, by using a Logiq S6 US system (GE^®^ Healthcare, Milwaukee, WI) and a 10–13 MHz broadband linear probe. We relied on the written reports of the ultrasound examinations to define the localization of the nodule rather than reviewing the images ourselves due to the retrospective design of the study.

US-guided FNAB had been performed by endocrinologists according to ATA guidelines [10]. American College of Radiology Thyroid Imaging and Recording Data System (ACR-TI-RADS) was used for classifying nodules in the US report [11]. Nodules were reported either in the lateral lobes (right and left) or isthmus. The thyroid gland was divided into three equal parts according to the longitudinal axis to determine the upper, middle and lower portions. The FNABs were performed with 25-gauge needles, and at least three samplings were performed for each nodule. The same experienced cytopathologist (K.C) reported the results according to The Bethesda System for Reporting Thyroid Cytopathology [12] and conducted the histopathological examination. All patients evaluated in the study underwent total thyroidectomy, and if necessary, central LN or lateral LN neck dissection for several reasons (pressure signs and suspicion or detection of malignancy). American Joint Committee on Cancer Tumour, Node, Metastasis cancer staging system eighth edition was used for staging thyroid malignancies. The maximal size of the metastatic LN foci smaller than 0.2 cm was approved as micrometastasis for LNs [11, 13].

The study was approved by the local Institutional Research Ethics Committee (Ankara University, I10-615-20) and was certified that the study was performed in accordance with the ethical standards of the 1964 Declaration of Helsinki.

### Statistical analysis

Descriptive statistics are summarized as counts and percentages for categorical variables; mean and standard deviations for normally distributed continuous variables and median (interquartile range) for non-normally distributed continuous variables or ordinal variables. The difference between the two groups was evaluated using the Student’s t test for normally distributed variables and the Mann Whitney U test for non-normally distributed variables. The differences between two groups in terms of categorical variables were compared by using Chi-Square test or Fisher’s exact tests, where applicable. Univariate logistic regression analysis was used to evaluate all possible risk factors. Variables with p < 0.20 in the univariate analysis were considered as candidate variables, along with all known clinically significant variables for the multiple logistic regression model. In order to define independent risk factors of the outcome variable, multiple logistic regression analysis was used and adjusted odds ratios were calculated. The odds ratios and their 95% confidence interval (CI) were calculated for each independent variable. Two-tailed p value less than 0.05 was considered significant.

## Results

A total of 9504 patients with thyroid US data and detailed nodule information (dimensions, presence of calcification, echogenicity and localization, among others) were obtained. A mean age of 55.2 ± 13.0 years with a female to male ratio of [7618 (80.2%)/1886 (19.8%)] were included in the study. A total of 962 (10.1%) nodules had an isthmic localization, whereas 8542 (89.9%) nodules had a lobar localization. According to nodule localizations, 4870 nodules (51.2%) were observed in the right lobe, 3672 nodules (38.6%) in the left lobe, and 962 nodules (10.1%) in the isthmus. In addition, 1220 nodules (17.4%) were observed in the upper portion, 3596 nodules (51.2%) in the middle portion, and 2204 nodules (31.4%) in the lower portion. The general characteristics of the thyroid nodules are shown in Table 1.

**Table 1:** The general characteristics of the thyroid nodules involved in the study

Age(years), mean ± SD		55.3 ± 13.0
Gender (F/M)* n (%)*		7618 (80.2%)/1886 (19.8%)
Localization of the nodule	*İsthmus n (%)*	962 (10.0%)
	*Lober n (%)*	8542 (90.0%)
Echogenity	*Hypoechoic n (%)*	3084 (32.4%)
	*Isoechoic n (%)*	6190 (65.1%)
	*Hyperechoic n (%)*	230 (2.4%)
Presence of macrocalcification	*Absent n (%)*	9131 (96.1%)
	*Present n (%)*	373 (3.9%)
Presence of microcalcification	*Absent n (%)*	8983 (94.5%)
	*Present n (%)*	521 (5.5%)
Irregular margins	*Absent n (%)*	451 (4.7%)
	*Present n (%)*	9053 (95.3%)
Structure of the nodule	*Solid n (%)*	3031 (39.8%)
	*Cystic n (%)*	987 (13%)
	*Mixed n (%)*	3600 (47.2%)
ACR-*TIRADS*	*1 n (%)*	196 (2.6%)
	*2 n (%)*	1122 (14.7%)
	*3 n (%)*	756 (9.9%)
	*4 n (%)*	4400 (57.8%)
	*5 n (%)*	1144 (15.0%)
Number of nodules	*Solitary n (%)*	4425 (46.6%)
	*Multiple n (%)*	5079 (53.4%)
Bethesda classification	*1 n (%)*	327 (3.4%)
	*2 n (%)*	6855 (72.1%)
	*3 n (%)*	1040 (10.9%)
	*4 n (%)*	169 (1.8%)
	*5 n (%)*	310 (3.3%)
	*6 n (%)*	803 (8.4%)
Maximum diameter of the nodule (cm)	*Isthmic nodules n (%)*	13 (0–80)/15,5 ± 9,4
	*Lobar nodules n (%)*	13 (0–99)/16,5 ± 12,1

*F* Female, *M* Male, *ACR-TIRADS* American College of Radiology Thyroid Imaging and Recording Data System

In our study, the differences between the nodules in isthmic and lobar localizations in terms of risk of malignancy, compositions, margins, Bethesda classifications and metastases patterns, among others, were investigated.

Approximately 49.1 and 46.3% of isthmic and lobar nodules were solitary (p = 0.100). Moreover, 2.0, 11.8, 9.6, 60.4%, and 16.2% of isthmic nodules were TR1, TR2, TR3, TR4 and TR5, respectively, according to TI-RADS scoring. Similarly, 2.6, 15.0, 10.0, 57.5, and 14.9% of lobar nodules were TR1, TR2, TR3, TR4 and TR5, respectively. Both 108 isthmic nodules (11.2%) and 695 lobar nodules (8.1%) were evaluated as Bethesda 6. In addition, 110 (11.4%) isthmic nodules and 930 (10.9%) lobar nodules were reported as Bethesda 3 (atypia of undetermined significance).

### Malignant nodules

A total of 1188 nodules were confirmed as malignant from histopathological evaluation. The mean age of the patients with malignant nodules was 49.5 ± 14.0 years. When excluding anaplastic thyroid carcinoma (because of advanced age), the mean age was 49.3 ± 13.9 years. The mean age of papillary thyroid cancer (PTC) was 49.0 ± 13.8 years, and the mean ages of follicular thyroid cancer (FTC), medullary thyroid cancer (MTC), Hurtle cell carcinoma and anaplastic thyroid carcinoma were 50.8 ± 14.1 years, 50.5 ± 14.0 years, 50.3 ± 12.0 years, and 72.4 ± 7.7 years, respectively.

Of the 1188 malignant nodules, 986 nodules (83.0%) were PTC, 114 nodules (9.6%) were FTC, 55 nodules (4.6%) were MTC, 16 nodules (1.3%) were Hurtle cell carcinoma, and 8 nodules (0.7%) were anaplastic thyroid carcinoma, and 9 nodules (0.8%) were thyroid tumors of uncertain malignant potential (TT-UMP). 156 of the malignant nodules (13.1%) were located in the isthmus, whereas majority of the malignant nodules (n = 1032, 86.9%) were located at the lobar parts (right or left) of the thyroid.

The distribution of lobar PTC, FTC, MTC, Hurtle cell carcinoma, and anaplastic cell carcinoma within the lobes were as follows: for lobar PTCs, 198 (26.7%) nodules were in the upper, 356 (48.0%) nodules were in the middle, and 187 (25.2%) nodules were in the lower zones of the thyroid; for lobar FTCs, 9 (13.6%) nodules were in the upper, 34 (51.5%) nodules were in the middle, and 23 (34.8%) nodules were at the lower zones of the thyroid; for lobar MTC, 4 (10.0%) nodules were in the upper, 32 (80.0%) nodules were in the middle, and 4 (10.0%) nodules were at the lower zones of the thyroid; for lobar Hurtle cell carcinoma, 2 (15.4%) nodules were in the upper, 10 (76.9%) nodules were in the middle, and 1 (7.7%) nodule was at the lower zones of the thyroid; and for lobar anaplastic carcinoma, 3 (75%) nodules were in the middle and 1 (25.0%) nodule was at the lower zone of the thyroid, respectively (p = 0.001).

### Tumour, node, metastasis stage

TNM staging was performed in 788 of the surgically removed nodules. 657 nodules (83.4%) were classified as T1 (1a,1b), 88 nodules (11.2%) as T2, 36 nodules (4.6%) as T3 (3a, 3b), and 7 nodules (0.9%) as T4 (4a, 4b). A total of 637 nodules (80.8%) were classified as N0 and 151 (19.1%) were classified as N1. All of the thyroid malignancies were M0. For thyroid cancers in the isthmic region, 93 (87.7%) were evaluated as stage T1 (1a, 1b), 10 (9.4%) as stage T2, and 3 (2.8%) as stage T3 (3a, 3b). No isthmic cancer was classified as T4. Furthermore, 83 (78.3%) of the isthmic cancers were classified as N0 and 23 (21.7%) as N1.

### Capsular, muscular invasion and LN metastases

When metastasis patterns were examined, capsular and muscle invasions were observed in 92/802 (11.5%) of the nodules and LN metastases were observed in 152/802 (19%) of the nodules. There was no significant difference in capsular invasion between isthmic (9/108; 8.3%) and lobar cancers (75/694; 10.8%) (p = 0.435). There was no significant difference in muscular invasion between isthmic (2/108; 1.9%) and lobar cancers (6/694; 0.9%) (p = 0.294).

Of the 152 LN metastases, there was no significant difference in lymph node metastasis in between isthmic (24/108; 22.2%) and lobar cancers (128/694; 18.4%) (p = 0.351). There was no lateral lymph node metastasis in isthmic cancers but, 13/128 (10.2%) lateral lymph node metastasis were observed in lobar cancers (p = 0.224).

As a summary, we reported US and FNAB data distinguishing between isthmic nodule vs non-isthmic nodules (Table 2).

**Table 2:** Distribution and TNM Staging of Thyroid Cancers by Location

Types of cancer	Isthmus	Lobar	p value
PTC	93/704 (13.2%)	611/704(86.8%)	** < 0.001**
MTC	5/35 (14.3%)	30/35(85.7%)	** < 0.001**
FTC	8/46 (17.4%)	38/46(82.6%)	** < 0.001**
Hurtle cell	1/14 (7.1%)	13/14(92.9%)	** < 0.001**
Anaplastic	1/4 (25%)	3/4(75%)	0.485
TNM staging			
T1 (1a and 1b)	93/657 (14.2%)	564/657(85.8%)	** < 0.001**
T2	10/88 (11.4%)	78/88(86.6%)	** < 0.001**
T3 (3a and 3b)	3/36 (8.3%)	33/36(91.7%)	** < 0.001**
T4 (4a and 4b)	0/7(0%)	7/7 (100%)	** < 0.001**
N0	23/151(15.2%)	128/151(84.8%)	** < 0.001**
N1	83/637(13%)	554/637(87%)	** < 0.001**
M0	108/788(13.7%)	680/788(86.3%)	** < 0.001**

Those with significant p values are labeled in bold

*PTC* papillary thyroid cancer, *MTC* Medullary thyroid cancer, *FTC* Follicular thyroid cancer, *TNM* Tumor, node, metastasis

In our univariate logistic regression analysis, a significant relation was found between age, nodule localization, nodule shape, nodule echogenicity, nodule composition, margin irregularity, microcalcification, macrocalcification, number of nodules, TI-RADS 5 and maximum diameter. In the multivariate logistic regression model, age, nodule localization and TI-RADS 5 continued to be significant risk factors for malignancy (Tables 3, 4).

**Table 3:** Univariate and multivariate logistic regression analysis for malignancy risk

	Univariate analysis			Multiple analysis		
	OR	95% CI	p	OR	95% CI	p
Age (years)	0.962	0.958–0.967	< 0.001	0.966	0.960–0.971	< 0.001
Sex (Male vs Female)	1.146	0.989–1.329	0.071			
Nodule localization						
Right lobe vs left lobe	1.220	1.068–1.394	0.003			
Isthmus vs left lobe	1.583	1.296–1.935	< 0.001			
Isthmus vs lobar	1.408	1.173–1.692	< 0.001			
Isthmus vs lower pole	1.763	1.413–2.200	< 0.001	1.594	1.214–2.093	0.001
Upper pole vs lower pole	1.938	1.581–2.375	< 0.001	1.950	1.541–2.467	< 0.001
Middle pole vs lower pole	1.264	1.064–1.500	0.008	1.346	1.105–1.641	0.003
Nodule shape						
L ≥ W vs W > L	1.225	1.051–1.429	0.010			
Nodule echogenicity						
Hypoechoic vs isoechoic and hyperechoic	2.162	1.912–2.446	< 0.001			
Nodule composition						
Solid vs cystic	1.388	1.060–1.816	0.017			
Mixed vs cystic	2.454	1.885–3.194	< 0.001			
Margin irregularity						
Irregular vs regular	8.706	7.154–10.595	< 0.001			
Microcalcification	5.231	4.335–6.313	< 0.001			
Macrocalcification	1.845	1.423–2.392	< 0.001			
Number of nodules						
Solitary vs Multinodular	1.345	1.191–1.519	< 0.001			
TI-RADS 5						
4 vs 1–2-3	1.058	0.878–1.274	0.554	1.105	0.907–1.347	0.321
5 vs 1–2-3	4.044	3.305–4.949	< 0.001	3.880	3.131–4.809	< 0.001
Maximum diameter (mm)	0.993	0.987–0.998	0.013			

*W* Width, *L* Length

**Table 4:** Univariate and multivariate logistic regression analysis for lymph node metastasis

	Univariate analysis			Multiple analysis		
	OR	95% CI	p	OR	95% CI	p
Age (years)	0.957	0.943–0.970	** < 0.001**	0.949	0.934–0.964	** < 0.001**
Sex (Male vs Female)	2.374	1.603–3.516	** < 0.001**	2.657	1.707–4.136	** < 0.001**
Nodule Localization						
Isthmus vs Lobar	1.263	0.772–2.067	0.352			
Upper pole vs other lobes	1.693	1.107–2.591	**0.015**	1.889	1.201–2.970	**0.006**
Number of Nodules						
Solitary vs Multinodular	1.208	0.848–1721	0.295			
TI-RADS 5						
5 vs 1–2-3–4	1.976	1.320–2.957	**0.001**			
Tumor Size						
T2 vs T1	1.613	0.963–2.702	0.069			
T3 vs T1	1.303	0.579–2.930	0.523			
T4 vs T1	3.419	0.755–15.481	0.111			

Those with significant p values are labeled in bold

## Discussion

Solid isthmic thyroid nodules were found to be less likely MTC; however, these nodules have a higher risk for thyroid cancer than patients with nodules located in the lateral lobes [6].

PTC is the most common thyroid malignancy, and most PTCs have an indolent course and an excellent prognosis. However, some PTCs with certain features are likely to recur, have distant metastasis and have a poor prognosis. Advanced age, male sex, large tumour size, advanced stage, multiplicity of the nodules, extrathyroidal extension, and presence of LN metastasis or distant metastasis at the time of diagnosis are the main factors affecting PTC prognosis. Although the incidence of PTC arising in the thyroid isthmus is low (1–9.2%), previous studies have shown that isthmic PTCs are more likely to be multifocal, have capsule invasion and extrathyroidal extension, and more likely involve the central LN [9, 14–17]. Thyroid malignant lesions located in the isthmus are more aggressive and associated with a poor prognosis [7, 18–20].

In addition to surgical studies showing that isthmic PTCs have a poor prognosis, in recent years, the location of the nodule within the gland has also been associated with a distinct risk of malignancy, with isthmic and upper lobar nodules presenting with the highest risk [8, 21, 22]. Although a localization relationship study was conducted to determine thyroid cancer frequency in people exposed to radiation, no significant distribution characteristic was detected in that study [23].

The first studies evaluating the risk of thyroid malignancy according to the anatomical site of thyroid nodules was published just a few years ago and suggested that the longitudinal location in the lobe may be a risk factor independent of US appearance, and thyroid nodules located in the upper pole can be considered as having a higher risk for malignancy [21, 22].

Subsequently, Jasim et al. reported a large cohort study consisting of 3419 nodules obtained from 3313 patients and demonstrated that the odds (OR: 2.4 [1.6–3.6], p < 0.0001) of a nodule being malignant is highest in the isthmus. In the multivariate regression model adjusting for age, sex, family history of thyroid cancer, radiation exposure, nodule size and ACR TI-RADS score, the risk of malignancy remained highest in the isthmus (OR: 2.4 [1.5–3.9], *p* = 0.0007), followed by upper thyroid nodules (OR: 1.8 [1.2–2.7], *p* = 0.005) and then middle thyroid nodules (OR: 1.5 [1.1–2.0], *p* = 0.01) compared to lower thyroid nodules[8].

We obtained similar results in our study for isthmic nodules. The major findings of our study include the following: (i) nodule localization is a risk factor for malignancy in addition to very-well-known sonographic criteria, and isthmic nodules have a similar risk for malignancy as in the upper zones (OR: 1.79 [CI 1.38–2.34] p < 0.001); (ii) localization is an independent risk factor for DTC (OR: 1.35%, 95% CI p = 0.01); (iii) there was no significant difference between isthmic and lobar malignant nodules in terms of the capsule, muscle, and LN metastasis; and (iv) although there was no significant difference observed between localizations in terms of micrometastasis, lobar cancers were found to have a higher risk for macrometastasis (OR: 8.64, 95% CI p = 0.04) than isthmic cancers. Based on the data of two-thirds of our patients for whom we have TNM staging information, a prophylactic central LN dissection in isthmic PCTs may not be required.

The rationale for the association between isthmic localization and cancer risk remains unclear, but some explanations were proposed such as the embryologic nature of the gland. The thyroid gland in humans is an organ formed by the fusion of three anlagen that develop from the anterior foregut [24]. It was suggested that, during embryology, the early formation of lobes would depend on the incorporation of the two lateral anlagen, and conversely, the isthmus would be a remnant of the median anlage, which could possibly reflect a different cellular composition and a higher associated risk of malignancy. Besides, the isthmus has less thyroid tissue compared with the lobes and therefore may be less prone to hyperplastic processes [25]. The middle and superior thyroid veins have a tortuous route, and this route leads to slower venous drainage of the upper lobes than the lower lobes. The relatively higher levels of reactive oxygen species locally in the tissue, which is involved in the regulation of telomerase activity through the Akt pathway and induction of cancer-promoting mutations, may induce carcinogenesis in the upper lobes and thyroid isthmus. Another explanation is the exposure of the upper poles of the thyroid gland to high doses of X-ray radiation during dental procedures or computed tomography head/neck scans [22]. Also, the cause for its multifocal nature, predisposition for capsule invasion or extrathyroidal extension, and central LN involvement for malignant isthmic nodules is due to the smaller and thinner size of this portion of the gland and its unique lymphatic drainage [25]. However, its aggressiveness has not been demonstrated in this study.

The strength of our study was primarily elucidating histopathological (assessed by a single pathologist) and US data (by the same experienced endocrinologists) of a larger number of nodules in a single centre over a long period. The limitation of our study was the retrospective design of the study which involved patients among a long period time and TIRADS ultrasound risk estimation was relied by US report and may be misleading. We relied on the written reports of the ultrasound examinations to define if a nodule was in the isthmus or a lobe, rather than reviewing the images ourselves. Uniformly reviewing the images would significantly increase the value of this study.

Specific guidelines for thyroid cancers arising in the isthmus are non-existent, and treatment for isthmus cancer may not be different from other localizations. Moreover, nodule localizations have not yet been included in the thyroid nodule guidelines as a risk factor; however, the increasing number of publications on this subject should be highlighted. Our study is the current retrospective design study with the highest number of thyroid nodules and examined the relationship between localization and risk of malignancy. We argue that a more detailed US (capsule invasion, extrathyroidal extension, and muscle invasion) should be performed for cytopathologically established malignant nodules with isthmic localization. However, also according to the retrospective design of our study other than the absence of both histological data (from one-third of our cohort of patients) and iodine-131 whole body scintigraphy results, a final suggestion for or against prophylactic central LN dissection cannot be suggested. Accordingly, pros and cons (i.e. for or against central LN dissection) should always be discussed in a local multidisciplinary team taking a final decision for each patient. We recommend nodule localization has to be considered an additional risk factor when performing a Fine Needle Aspiration Biopsy for the increased malignancy risk in this localization.

## Data Availability

The data that support the findings of this study are available on request from the corresponding author. The data are not publicly available due to privacy or ethical restrictions.
